# Changes in Rectal Dose Due to Alterations in Beam Angles for Setup Uncertainty and Range Uncertainty in Carbon-Ion Radiotherapy for Prostate Cancer

**DOI:** 10.1371/journal.pone.0153894

**Published:** 2016-04-20

**Authors:** Yoshiki Kubota, Hidemasa Kawamura, Makoto Sakai, Ryou Tsumuraya, Mutsumi Tashiro, Ken Yusa, Nobuteru Kubo, Hiro Sato, Masahiro Kawahara, Hiroyuki Katoh, Tatsuaki Kanai, Tatsuya Ohno, Takashi Nakano

**Affiliations:** Gunma University Heavy Ion Medical Center, Gunma, Japan; Taipei Medical University, TAIWAN

## Abstract

**Background and Purpose:**

Carbon-ion radiotherapy of prostate cancer is challenging in patients with metal implants in one or both hips. Problems can be circumvented by using fields at oblique angles. To evaluate the influence of setup and range uncertainties accompanying oblique field angles, we calculated rectal dose changes with oblique orthogonal field angles, using a device with fixed fields at 0° and 90° and a rotating patient couch.

**Material and Methods:**

Dose distributions were calculated at the standard angles of 0° and 90°, and then at 30° and 60°. Setup uncertainty was simulated with changes from −2 mm to +2 mm for fields in the anterior-posterior, left-right, and cranial-caudal directions, and dose changes from range uncertainty were calculated with a 1 mm water-equivalent path length added to the target isocenter in each angle. The dose distributions regarding the passive irradiation method were calculated using the K2 dose algorithm.

**Results:**

The rectal volumes with 0°, 30°, 60°, and 90° field angles at 95% of the prescription dose were 3.4±0.9 cm^3^, 2.8±1.1 cm^3^, 2.2±0.8 cm^3^, and 3.8±1.1 cm^3^, respectively. As compared with 90° fields, 30° and 60° fields had significant advantages regarding setup uncertainty and significant disadvantages regarding range uncertainty, but were not significantly different from the 90° field setup and range uncertainties.

**Conclusions:**

The setup and range uncertainties calculated at 30° and 60° field angles were not associated with a significant change in rectal dose relative to those at 90°.

## Introduction

As compared with photon beams, particle beams provide sharper dose distributions by taking advantage of the Bragg peak and a sharp lateral penumbra [1]. Avoiding excessive exposure to organs at risk (OARs) requires understanding of the influences of setup error and of beam range error.

In particle therapy for prostate cancer, 90° horizontal fields are often used to reduce the rectal dose. It is easy to reduce the rectal dose by collimation without considering changes in the specific range of the particle beam, because small changes in range do not significantly affect the dose. However, horizontal fields are contraindicated after hip surgery employing metal plates or prostheses because of the unpredictability of the beam’s path through the metal and the influence of artifacts. For example, Jäkel et al. reported that in the case of tungsten and steel, metal path range errors of −5% and −18%, respectively, were observed, together with 1% path range errors involving the artifacts from titanium and steel [2]. Although artifact unpredictability was small if the metal was light, path unpredictability through the metal was large. Therefore, it is preferable to use 0° vertical (perpendicular to the patient body surface) or oblique fields in these cases. The oblique field might have a different sensitivity to the horizontal field regarding inaccuracies in patient setup and beam range; however, its influence is not well defined. Tang et al. and Christodouleas et al. reported a comparison of dose distribution in the anterior-oriented fields used for proton therapy; but, they did not consider the uncertainties involved [3,4]. Inter/intra fractional motion changes of the prostate could have an effect. However, only the influences of inaccuracies in setup and beam range were evaluated in this study. Although cases involving cancer patients with metal implants are not frequent, it is important to determine their influence on the oblique fields, because this has the potential to reduce uncertainty regarding the rectal dose using the current treatment.

The polybinary calibration method between CT density value and effective density for particle beam radiotherapy has an accuracy of 99% [5,6]. The resulting beam range uncertainties cause dose deviations that can result in errors in dose to the clinical target volume (CTV) and OARs located along or near the beam path. OARs located lateral to the target may be exposed to higher doses as a result of setup errors. Although robust optimizations of treatment planning including setup and range uncertainty for proton therapy have been proposed [7,8], the influences of dose distribution per field angle have not been considered.

We evaluated the influence of setup and range uncertainties on the rectal and CTV dose distribution of oblique fields as compared with a horizontal (90°) field in prostate cancer. Although the bladder dose might also change for each field angle, the rectal dose was focused in our study to simplify the problem because the bladder is unlikely to be a clinical problem.

## Materials and Methods

### Patients

We retrospectively studied the data of ten prostate cancer patients aged 59–74 years with a median age of 69.5 years. Three patients had a titanium metal hip implant. These were located on the left side in two patients and on the right side in one patient; seven patients did not have hip implants. The CTV includes the prostate and proximal seminal vesicle (SV), and rectal volume measured from CT images was 18.0–97.2 cm^3^ and 48.5–84.7 cm^3^ with medians of 36.1 cm^3^ and 70.7 cm^3^, respectively. Patient information, CTVs, and rectal volumes are detailed in **Table 1**. This study was approved by the Institutional Review Board at Gunma University Hospital (approval number: 1310), and patient records/information were anonymized and de-identified prior to analysis.

**Table 1:** Patient information, CTVs, and rectal volumes. CTV shows the clinical target volume, metal implant shows which side the patient has in or not.

Patient Number	Age	CTV [cm^3^]	Rectal Volume [cm^3^]	Metal Implant
P1	70	18.0	48.5	Left
P2	72	18.2	69.1	Right
P3	74	21.9	77.8	Left
P4	69	44.6	65.9	Non
P5	59	97.2	58.7	Non
P6	59	38.5	69.4	Non
P7	70	53.3	72.1	Non
P8	61	22.9	84.7	Non
P9	61	41.0	80.8	Non
P10	72	33.8	83.4	Non
Median	69.5	36.1	70.7	

Metal implant refers to whether the patient has a metal implant and on which side.

### Irradiation devices and treatment planning

The Gunma University Heavy Ion Medical Center (GHMC) provides carbon-ion therapy [9] using a heavy ion irradiation device (Mitsubishi Electric, Tokyo, Japan) with a passive irradiation method [10] and a treatment planning system (TPS) (XiO-N, Mitsubishi Electric). The passive irradiation field was generated using the scatterer and wobbling, and the field was collimated to the outside of the PTV using a multi-leaf collimator (MLC). X-ray CT (Acquilion LB, Self-Propelled, Toshiba Medical Systems, Tochigi, Japan) images were acquired with non-helical, 2.0 mm × 4 acquisitions, full reconstruction mode, and pixel spacing was 1.07 × 1.07 mm. The average number of CT slices for prostate cancer patients was approximately 140. XiO-N incorporates a dose engine for ion beam radiotherapy dose calculations (K2-Dose) [11–14]. The relative biological effectiveness (RBE) was included in the absorbed dose using a spread-out Bragg peak concept [15], and the clinical dose including this was defined as Gy(RBE). This RBE concept was incorporated into the XiO-N. The planning target volume (PTV) for prostate cancer was created by adding the anterior and lateral margins of 10 mm, cranial and caudal margins of 6 mm, and a posterior margin of 5 mm to the CTV, but lateral margins to the proximal SV were 10 mm. Carbon ion treatment plans were generated as each PTV was covered with 95% of the prescribed dose. In treating prostate cancer, we used five fields, and the number of fractions for each field was normally 3, 3, 3, 4, and 3 or 3, 3, 3, 3, and 4 (16 fractions total). Thus, for one fraction, we used 3.6 Gy(RBE);and the total dose was 3.6 × 16 = 57.6 Gy(RBE).

**Fig 1:**
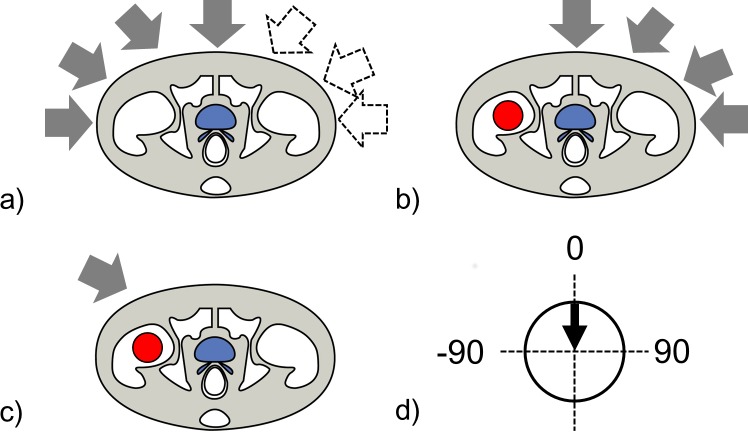
The two patterns of CT image sets used for the calculation. Arrows show the beam directions, blue regions show the CTV, and red regions show the metal implant. (a) Diagram of a patient with no implant and a beam that can enter from the left (negative angle, gray arrows) or the right (positive angle, white arrows). (b) Diagram of a patient with a hip implant, showing the field directions of 0°, 30°, 60°, and 90°. (c) Diagram of a patient with a hip implant, showing the oblique field avoiding the implant. (d) Field directions from −90° to 90°; 90° represents left horizontal, and −90° represents right horizontal.

In this planning study, the two patterns of CT image sets shown in **Fig 1** were used for the calculation of dose distribution to evaluate the influence of dose deviations in each field angle, and to evaluate the actual fields used for the treatment. The first pattern was seven CT datasets for patients that had no implants as shown in **Fig 1(A)**, and three CT datasets for patients with hip implants but with the opposite side of the implant as shown in **Fig 1(B)**. Four different field angles (0°, 30°, 60°, and 90°) in each image set were used, with the patient couch rotated accordingly; the beam parameters used in the planning for each field angle are detailed in **Table 2**. The second pattern was three CT image sets for patients with implants, using oblique fields as shown in **Fig 1(C)**. The field angles used for P1, P2, and P3 in treatment planning were 60°, 67.8°, and −35°, respectively. A prescription dose in all of the directional fields shown in **Fig 1(A), 1(B)** and **1(C)** was set to 10.8 Gy(RBE), corresponding to three fractions per field.

**Table 2:** Beam parameters used in calculations for treatment planning for each field angle (n = 10).

			Maximum MLC size [mm]		WEL to IC [mm]
Angle (degrees)	Beam Energy [MeV/n]	SOBP width [mm]	Width	Height	
0	290, 380	50–80	77.3±6.4	64.1±9.6	92.7±7.3
30	290, 380	50–80	74.2±7.6	64.1±9.6	101.2±7.5
60	380	60–90	69.8±10.0	64.1±9.6	136.8±9.6
90	380, 400	65–90	62.6±9.7	64.1±9.6	179.8±7.2

Parameters were beam energy, SOBP width, maximum multi-leaf collimator (MLC) size, and water equivalent path length from the patient surface to the isocenter (WEL to IC). Beam energy values were from 10 patients, SOBP width values represent the range for 10 patients, and maximum MLC size and WEL to IC values represent the mean and standard deviation for 10 patients.

### Creating setup uncertainty

In patient positioning, both orthogonal (frontal and lateral) X-ray images and digitally reconstructed radiographs from CT images are used, with the bony structures for landmarks [16]. We employed a 2-mm setup tolerance [17]. The QA mode of the TPS was used to evaluate the results of setup uncertainty, calculating the dose distributions after moving the field center from -2 mm to 2 mm in anterior-posterior (AP), left-right (LR), and cranial-caudal (CC) directions. The dose calculations for the evaluation of setup uncertainty were performed in four field angles for seven patients as shown in **Fig 1(A)**, in four field angles for three patients as shown in **Fig 1(B)**, and in each field angle for three patients as shown in **Fig 1(C)**.

### Creating range uncertainty

The stopping power ratios of the planning volumes were calculated with the polybinary calibration method using the CT density measurement/stopping power ratio [5,6]. The K2 dose used the ratio for calculating dose. Given the 99% accuracy of this method, we evaluated the uncertainty range using the following equation:

$$R_{uncertainty}=\sqrt{R_{Body}^{2}+R_{Beam}^{2}}$$ (1)

where *R*_Body_ is the alteration in path through the patient’s body estimated from the range uncertainty, and *R*_Beam_ is the alteration in the path the carbon beam travels before hitting the patient’s body surface. In this planning study, *R*_Body_ was set to 2% of a water equivalent path length from the patient surface to the isocenter (IC) and *R*_Beam_ was set to 1 mm from the specifications of our accelerator. The dose distributions with the range uncertainty were recalculated by changing the parameters of the range shifter (RSF) in four field angles for seven patients as shown in **Fig 1(A)**, in four field angles for three patients as shown in **Fig 1(B)**, and in each field angle for three patients as shown in **Fig 1(C)**.

### Estimation of setup and range uncertainties

Setup and range uncertainties were simulated by simultaneously changing the field center (along the worst-case direction in the AP, LR, and CC directions) and RSF parameters to construct a worst-case scenario; their dose distributions were calculated in four field angles for seven patients as shown in **Fig 1(A)**, in four field angles for three patients as shown in **Fig 1(B)**, and in each field angle for three patients as shown in **Fig 1(C)**. A calculated case without considering setup and range uncertainties was defined as a normal-case, the highest mean rectal dose was defined as the worst-case, and the lowest mean rectal dose was defined as the best-case in each combination of the setup and range uncertainties.

### Evaluation method

To evaluate the influence of the dose deviations due to the uncertainties in each field angle, we used a mean dose increase ratio *R*_Inc_ defined as

$$R_{Inc}=\frac{D_{mean,W}-D_{mean,N}}{D_{mean,N}}\times 100,$$ (2)

where *D*_mean,*N*_ is the mean rectal dose in the normal scenario, and *D*_mean,*W*_ is the mean rectal dose in the worst-case scenario.

Additionally, to evaluate the rectal dose volumes as a result of the uncertainties in each field angle, we used rectal 10, 50, and 95% volumes regarding the prescription dose (defined as V_10_, V_50_, and V_95_) in the normal, best, and worst cases for each field angle.

The *R*_Inc_ results for setup uncertainty and for range uncertainty were analyzed using the Wilcoxon test, and both of the *R*_Inc_ results for setup and range uncertainties and the results of the rectal dose volumes in the normal case, were analyzed using the Shapiro–Wilk normality test to determine if data were normally distributed, and using Dunnett’s multiple test. The level of statistical significance in the Wilcoxon and Dunnett’s multiple tests was set to 5%.

## Results

**Fig 2:**
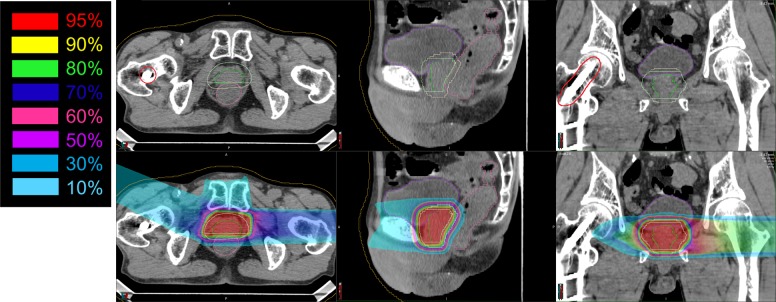
Clinical case data of a patient with a metal implant. The upper row shows CT images and the bottom row shows CT images together with the dose distribution. Left column shows axial images, middle column shows sagittal images, and right column shows coronal images. Red lines show the metal implant after hip replacement. Green line shows prostate, light yellow line shows PTV, magenta line shows rectum, and purple line shows the bladder.

The dose distribution of one case with a right hip implant is shown in **Fig 2**. For this patient treatment was as follows: three fractions using vertical fields, three fractions using horizontal fields from the left, two fractions using −67.8° fields from the right, five fractions using horizontal boost fields from the left, and two fractions using −67.8° boost fields from the right. All doses were 3.6 Gy(RBE) per fraction.

**Fig 3:**
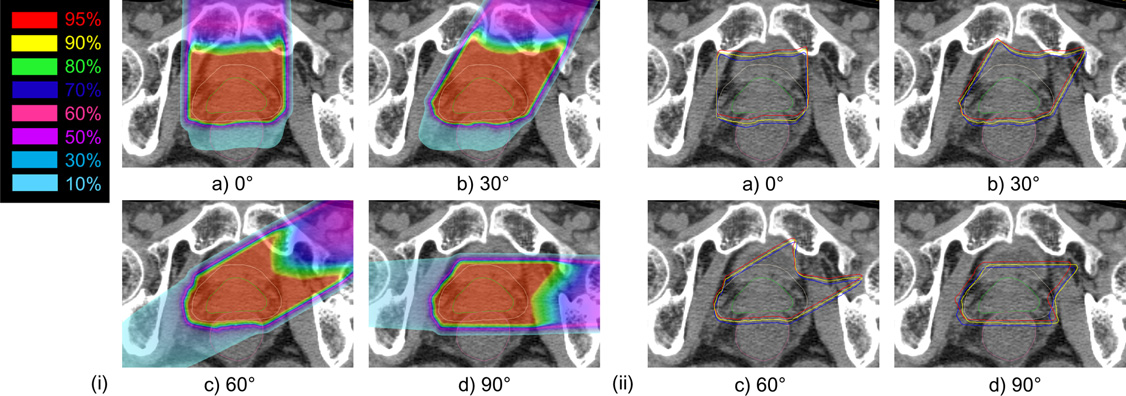
Data on one patient without a metal implant, for four field angles: (a) 0° field, (b) 30° field, (c) 60° field, and (d) 90° field. Green line shows prostate, light yellow line shows PTV, and magenta line shows rectum. (i) Dose distribution in the normal case. (ii) Yellow line shows the 95% isodose line for the prescription dose in the normal case, blue line shows the 95% isodose line of the prescription dose in the worst case, and red line shows the 95% isodose line of the prescription dose in the best case.

**Fig 4:**
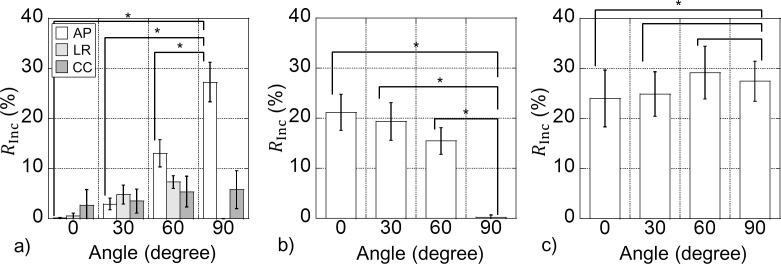
Increasing ratio graph of rectal mean dose from each uncertainty for the 10 patients enrolled in the study. (a) is the increasing ratio from setup uncertainty in anterior-posterior (AP), left-right (LR), and cranial-caudal (CC) directions, (b) is the ratio from range uncertainty, and (c) is the ratio from the setup and range uncertainties. The error bars represent the standard deviations for 10 patients. * in (a), and (b) show *p* < 0.05 using the Wilcoxon test, and * in (c) shows *p* < 0.05 using Dunnett’s multiple test.

Dose distributions for four field angles in one patient without the implant are shown in **Fig 3**. *R*_Inc_ graphs from the uncertainties for ten patients are shown in **Fig 4**.

**Fig 5:**
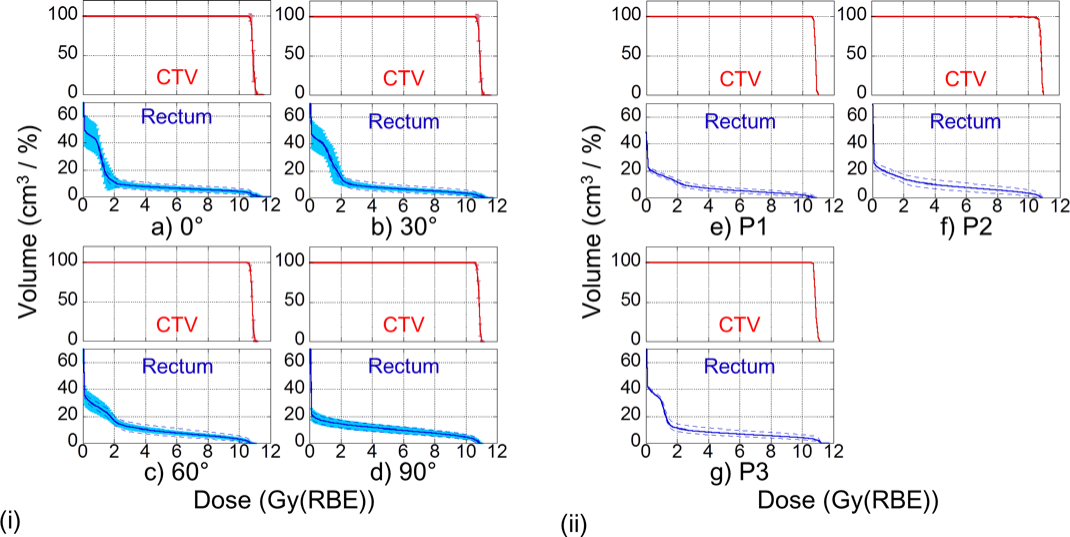
Averaging dose volume histogram (DVH) graphs of rectal dose and CTV dose. Red lines are the DVHs of the CTV dose shown as relative volume (%) and blue lines are DVHs of the rectal dose shown as absolute volume (cm^3^). (i) Ten patients in each beam angle. The light blue error bars represent the standard deviations for 10 patients. (ii) (e) is patient 1 with a 60° field. (f) is patient 2 with a 68° field. (g) is patient 3 with a −35° field. Patients in (e), (f) and (g) have hip implants and all fields avoid the implants. The solid lines show normal cases, and the dashed lines show the best or worst cases for setup and range uncertainties.

Dose volume histogram (DVH) graphs for the rectal dose and the CTV from setup and range uncertainties for ten patients, and DVH graphs in three patient cases with hip implants (**Fig 1(C)**) are shown in **Fig 5**; V_10_, V_50_, and V_95_ in normal, best, and worst cases for each field angle are shown in **Table 3**. In the normal cases (P1, P2, and P3) with the implant (**Fig 1(C)**), the V_10_ was 16.2 cm^3^, 18.4 cm^3^, and 25.7 cm^3^, respectively; the corresponding V_50_ was 5.5 cm^3^, 8.2 cm^3^, and 7.1 cm^3^, respectively, and the corresponding V_95_ was 1.4 cm^3^, 2.6 cm^3^, and 3.6 cm^3^, respectively. Additionally, *R*_Inc_ from the setup and range uncertainties for P1, P2, and P3 with the implant shown in **Fig 1(C)** was 25%, 33.1%, and 24.1%, respectively.

**Table 3:** Rectal dose volumes for 10 patients involving each beam angle in the normal, best, and worst cases. The values are the mean and standard deviation for 10 patients.

Angle (degrees)	V_10_ [cm^3^]			V_50_ [cm^3^]			V_95_ [cm^3^]		
	Normal	Best	Worst	Normal	Best	Worst	Normal	Best	Worst
0	33.4±7.5*	30.8±7.9	35.4±7.8	6.5±1.4*	3.9±1.1	9.5±1.8	3.4±0.9	1.4±0.6	5.7±1.3
30	35.4±7.8*	33.5±8.4	37.1±7.6	6.7±1.3*	4.0±1.1	9.9±1.5	2.8±1.1	0.9±0.8	5.2±1.2
60	26.1±5.2*	23.3±4.9	29.1±5.4	8.3±1.3*	5.3±1.4	11.7±1.5	2.2±0.8*	0.6±0.5	4.6±0.8
90	16.1±3.2	13.1±2.7	18.9±3.7	10.1±2.1	7.4±1.7	12.6±2.5	3.8±1.1	1.9±0.9	5.8±1.3

* shows a significant difference (*p*<0.05) from the 90° field in normal cases.

## Discussion

### The influences of field angles on rectal dose

The dose profile of the 0° field is affected by the depth and direction of the field, the dose profile of the 90° field is affected by the lateral direction of the field, and the dose profile of the 30° and 60° fields are affected by both depth and lateral directions. Considering the 0° fields, the rectum posterior to the PTV, is affected by the distal dose fall-off of the spread-out Bragg peak. Therefore, V_10_ from the 0° field was significantly larger than V_10_ from the 90° field angles shown in **Table 3** because of the distal tail. With the 90° fields, the rectum, lateral to the PTV for the field’s eye view, is affected by the lower lateral penumbra dose. The 30° and 60° fields increase the dose to the rectum by both effects. Therefore, the V_50_ from the 90° field is significantly larger than the V_50_ from the other field angles shown in **Table 3** because of the lateral penumbra dose. Additionally, the V_95_ from the 90° field is larger than the V_95_ from the other field angles detailed in **Table 3**, because the 90° field cannot deform into a re-entrant form of PTV on the beam’s path through. However, there were no significant differences from the 90° field to the 0° and 30° fields, but there was a significant difference between the 90° and 60° fields. Rucinski et al. reported that the V_70_ and V_90_ in the 90° field were respectively 12.2±4.7 cm^3^ and 5.9±2.6 cm^3^ for carbon beams [18], and Weber et al. reported the V_50 Gy_ in the 90° field was 19.3±3.1% for proton beams [19]. Our results in the 90° field were similar.

Tang et al. reported the rectal volume changes from the prescription dose in 0°, 30°, and 90° fields for proton beams [3]. Kraft and Bassler et al. reported that the lateral penumbra of the carbon beams is sharper than the lateral penumbra of the proton beams, and that the distal tail doses of the carbon beams are higher than the distal tail doses of the proton beams [1, 20]. Using these results, V_10_, V_50_, and V_95_ in each field angle are considered. As compared with the V_10_ for the carbon beams, V_10_ in the 90° field for the proton beams is higher than V_10_ in 0° and 30° fields. The cause is assumed to be that the distal tail of the proton beams is lower than the tail of the carbon beam. Both the V_50_ and V_95_ in the 90° field for the proton beams are lower than both the V_50_ and V_95_ in the 0° and 30° fields, similar to the fact that both the V_50_ and V_95_ in the 90° field for the carbon beams are lower than both the V_50_ and V_95_ in the 0° and 30° fields; however, the differences for the proton beams are larger than the differences for the carbon beams. The causes are assumed to be that the lateral penumbra for the proton beams is larger than the penumbra for the carbon beams, and the lateral penumbra for the proton beams in the 90° field beams results in increasing V_50_ and V_95_.

### The influence of separate setup or range uncertainties on rectal dose

Considering *R*_Inc_ from the setup uncertainty presented in **Fig 4**, *R*_Inc_ in the AP direction for a 90° field is significantly higher than the ratio for 0°, 30°, or 60°, and *R*_Inc_ in the LR and CC directions is lower than the ratio in the AP direction for 60° and 90° field angles. These findings indicate that the 90° field is disadvantageous for setup uncertainty, and the worst case for the setup uncertainty in the 90° field angle might be concerned only with the setup error in the CC direction. Additionally, *R*_Inc_ on 90° from range uncertainty is significantly lower than the ratio at 0°, 30°, and 60°. This shows that the 90° field is advantageous regarding range uncertainty.

### The influence of simultaneous setup and range uncertainties on rectal dose

Considering the influence of both setup and range uncertainties in **Fig 4**, *R*_Inc_ at 90° shows no statistically significant difference from the ratio for 30° or 60°. However, the 0° field was significantly lower than the ratio for 90°. This suggests that the 0° fields are less affected by uncertainty than fields at different angles. Meanwhile, there are some differences for the forms between DVHs; however, rectal dose increases for the 30° and 60° fields were almost the same as the dose increase for the 90° field. Therefore, the oblique fields can be safely used after checking the dose distribution and DVH. In particular, the V_95_ regarding the worst case for all angles was similar; however, the V_50_ was 0°<30°<60°<90°, and the V_10_ was 0°≈30°>60°>90° (**Table 3**). Therefore, the 0° field can be used to reduce the middle dose to the rectum, the 90° field can be used to reduce the low dose to the rectum, and the oblique fields can be used to reduce the middle dose and the low dose averagely. Additionally, the sensitivity of the applied irradiation procedure to the setup and range uncertainties is substantially limited because the standard deviations of V_10_, V_50_, and V_95_ were low.

However, in treatment planning, adjusting the rectal dose is achieved as follows: for the 0° field by altering the bolus; for the 90° field by altering the MLC; and for the 30° and 60° fields by altering both. Therefore, planning for the 30° and 60° fields is more complicated than for the 0° and 90° fields.

In the current study, the dose changes from setup and range uncertainties were evaluated using this simple realistic model, and the bladder dose was not evaluated. Although the bladder dose is not a clinical problem, changes in bladder capacity should be noted because they are sensitive to the beam range changes. It is valid not only for the passive irradiation method but also for an active irradiation method. It was useful for clinical treatment; however, we did not factor in dose changes during or between fractions [21–23]. If we assume that position changes regarding prostate intra/inter fraction motion contribute to setup uncertainty, we could use the setup uncertainty for the oblique field and the horizontal field accordingly. However, in the future a more extensive study will be necessary because the results of the current study were limited to a few patient cases and the study was performed using a specific treatment planning procedure.

### The influence on CTV dose from simultaneous setup and range uncertainties

There was no influence on dose to the CTV from setup and range uncertainties. We set PTV margins to the CTV in each direction as described in the Materials and Methods section. For example, the posterior margin does not protect against the range of uncertainties when the horizontal field is applied, but it does for the vertical field. In contrast, the posterior margin does not protect from the setup uncertainties when the vertical field is applied, but it does for the horizontal field. Taking into account the various factors, margins in all directions to the CTV are necessary to guarantee sufficient CTV coverage. Additionally, the CTV coverage will be guaranteed when the applied PTV margins widely exceed the shifts in the AP, LR, and CC directions.

### The evaluation of oblique fields avoiding the implants

The DVH forms shown in panels **(e)** and **(f)** in **Fig 5(ii)** are similar to the DVH form of averaging 60° shown in panel **(c)** in **Fig 5(i)**; the DVH form shown in panel **(g)** in **Fig 5(ii)** is similar to the DVH form of averaging 30° in **Fig 5(B)(i)**. Additionally, as compared with the V_10_, V_50_, and V_95_ for similar field angles of 30° (35.4±7.8 cm^3^, 6.7±1.3 cm^3^, and 2.8±1.1 cm^3^, respectively) or 60° (26.1±5.2 cm^3^, 8.3±1.3 cm^3^, and 2.2±0.8 cm^3^, respectively), the V_10_, V_50_, and V_95_ for P1, P2, and P3 (16.2 cm^3^, 5.5 cm^3^, and 1.4 cm^3^, respectively for P1; 18.4 cm^3^, 8.2 cm^3^, and 2.6 cm^3^, respectively for P2; and 25.7 cm^3^, 7.1 cm^3^, and 3.6 cm^3^, respectively for P3) were similar or lower, as detailed in **Table 3**. Furthermore, *R*_Inc_ values from the setup and range uncertainties were similar, as shown in **Fig 4**. Therefore, this demonstrates that oblique fields avoiding the implant could be safely used in the same way on the non-implant side. In treatment planning, the oblique fields were used to avoid the metal implant while remaining as close to the horizontal as possible. The oblique fields were as good as the horizontal fields in terms of the uncertainties. However, vertical fields might be better than oblique and horizontal fields, as shown in **Fig 4**.

## Conclusion

The influences of setup and range uncertainties on dose deviations in vertical, horizontal, and oblique fields were evaluated in this study. For the basic effect on the rectal dose, it was found that the vertical field could reduce the middle dose to the rectum, the horizontal field could reduce the low dose to the rectum, and the oblique fields could reduce the middle dose and the low dose averagely relative to the other fields. Additionally, the rectal dose deviations from the uncertainties in oblique fields showed no significant difference from those of the horizontal fields; it was found that oblique fields avoiding metal implants could be safely employed because the deviations did not increase with increasingly oblique field angles. The dose to the CTV was preserved over all obliquities.

Because robust optimization methods for the correction of uncertainties have been developed in intensity modulated radiation therapy [24,25], similar methods are needed in particle beam therapy. We hope our findings are the beginning of that process.
